# An evidence-based approach to identify aging-related genes in *Caenorhabditis elegans*

**DOI:** 10.1186/s12859-015-0469-4

**Published:** 2015-02-07

**Authors:** Alison Callahan, Juan José Cifuentes, Michel Dumontier

**Affiliations:** Stanford Center for Biomedical Informatics Research, School of Medicine, Stanford University, Stanford California, AC USA; Molecular Bioinformatics Laboratory, Millennium Institute on Immunology and Immunotherapy, 49 Santiago, CP 8330025 Portugal; Departamento de Genética Molecular y Microbiología, Facultad de Ciencias Biológicas, Pontificia Universidad Católica de Chile, Alameda 340, Santiago, Chile

**Keywords:** Aging, Lifespan, C. elegans, Semantic web, Linked data, SPARQL, SPARQL inferencing notation, Data integration, Hypothesis evaluation

## Abstract

**Background:**

Extensive studies have been carried out on *Caenorhabditis elegans* as a model organism to elucidate mechanisms of aging and the effects of perturbing known aging-related genes on lifespan and behavior. This research has generated large amounts of experimental data that is increasingly difficult to integrate and analyze with existing databases and domain knowledge. To address this challenge, we demonstrate a scalable and effective approach for automatic evidence gathering and evaluation that leverages existing experimental data and literature-curated facts to identify genes involved in aging and lifespan regulation in *C. elegans*.

**Results:**

We developed a semantic knowledge base for aging by integrating data about *C. elegans* genes from WormBase with data about 2005 human and model organism genes from GenAge and 149 genes from GenDR, and with the Bio2RDF network of linked data for the life sciences. Using HyQue (a Semantic Web tool for hypothesis-based querying and evaluation) to interrogate this knowledge base, we examined 48,231 *C. elegans* genes for their role in modulating lifespan and aging. HyQue identified 24 novel but well-supported candidate aging-related genes for further experimental validation.

**Conclusions:**

We use semantic technologies to discover candidate aging genes whose effects on lifespan are not yet well understood. Our customized HyQue system, the aging research knowledge base it operates over, and HyQue evaluations of all *C. elegans* genes are freely available at http://hyque.semanticscience.org.

**Electronic supplementary material:**

The online version of this article (doi:10.1186/s12859-015-0469-4) contains supplementary material, which is available to authorized users.

## Background

The biology of aging is a significant area of biomedical research, motivated by a desire to uncover the mechanisms that govern aging and control these processes in an effort to develop effective therapies for aging-related diseases. Experiments in model organisms have identified genes, gene variations, and biological pathways that regulate longevity [1] in humans [2,3] and model organisms such as the nematode *Caenorhabditis elegans* [4], and mutations in many of the genes responsible for regulating longevity in model organisms are implicated in human disease [5,6]. Genetic manipulations that extend lifespan have been found to simultaneously abolish many degenerative effects that are the hallmarks of aging and aging-related disease [1], indicating that the biological pathways underlying these phenotypes are closely linked. Environmental factors such as dietary restriction [7], temperature [8,9] and pheromone exposure [10] have also been found to have significant effects on lifespan in model organisms, often acting through stress response genes and pathways whose activity are triggered by changes in nutrient availability [1].

Biologists studying the role of genes in aging use a variety of approaches, and a typical experiment involves perturbing environmental conditions or gene expression in vivo, measuring changes in lifespan, and measuring associated changes in gene expression to identify potential genetic agents mediating observed lifespan effects. The use of high-throughput experimental techniques such as microarrays and next generation sequencing platforms capable of measuring changes in expression of thousands of genes, combined with the large body of existing experimental data, publications and databases dedicated to capturing aging-associated annotations makes it increasingly intractable for scientists to manually sift through these resources. Large-scale bioinformatics analyses of genes and aging pathways have seen recent success in identifying candidate aging-related genes and elucidating their expressed products’ effects on biological pathways [11-14], and the fruits of this labor are increasingly accessible by the scientific community. For example, the Human Aging Genomics Resources group maintains the GenAge and GenDR databases of both model organism and human genes and their experimentally determined effects on lifespan and aging. GenAge contains human curated annotations of human and model organism genes that are known to affect aging, as well as candidate aging genes. GenAge annotations include the influence of the gene on longevity, the maximum average lifespan change associated with the gene, known molecular functions of gene products, and links to the literature. GenDR contains curated data on genes that modulate longevity by affecting lifespan extension conferred by dietary restriction (DR), and on genes whose expression is changed by DR. Model organism databases such as WormBase also capture gene and phenotype annotations related to aging and lifespan. Such resources will continue to grow in both size and span as more experimental data is generated.

With the increased availability of data about aging-related phenomena, a significant challenge lies in finding, integrating, and evaluating these data sources to address questions of biological interest, in this case in discovering genes that are responsible for the biological processes that govern aging and longevity. Ontologies and linked data, and technologies for creating them including the Web Ontology Language (OWL) and the Resource Description Framework (RDF) can enable such applications by allowing scientists to represent data and knowledge in a machine understandable way, such that we can leverage computational power to query and reason over them [15,16]. An ontology is the specification of a conceptualization [17] that describes concepts in a domain of knowledge and the relations between them. Linked Data is a paradigm for publishing data on the Web that uses RDF as a foundation for representation, and aims to make data a first class citizen of the Web to enable its widespread sharing, integration and re-use [18]. More specifically, life sciences data on the Semantic Web such as Bio2RDF [19,20] and the growing number of bio-ontologies enable data integration and powerful question answering in a variety of biological and biomedical domains [21-23]. Motivated by these developments, we use HyQue [24,25] to evaluate hypotheses on the roles of *C. elegans* genes in aging. HyQue is a Semantic Web tool that uses W3C standards (RDF/OWL) for representing data, domain-specific knowledge (in ontologies) and evaluation rules to computationally evaluate biological hypotheses over existing data resources. In this work, we customized HyQue by developing aging domain-specific hypothesis evaluation rules and used the custom system to execute them in a single pass over all *C. elegans* genes.

Two areas of related research are systems for hypothesis generation and evaluation, and the application of predictive methods for discovering aging-related genes. In the first area, there have been several research efforts focused on formulating and representing hypotheses and computationally evaluating hypotheses using existing data. HypGene [26,27], implemented in Lisp, was designed to describe and evaluate hypotheses about genetic attenuation by using theory revision operators to iteratively update hypotheses about the trp operon based on experimental data. HinCyc [28] was a tool that used data about *E. coli* pathways in the EcoCyc encyclopedia of *E. coli* genes and pathways [29] to hypothesize the occurrence of similar pathways in *H. influenzae*. GenePath [30], a system implemented in Prolog, used abductive reasoning and if-then rules to generate hypotheses about genetic networks based on genetic experiments in *D. discoideum*. Adam the Robot Scientist [31] is a combination system for carrying out automated wet lab experiments and formulating hypotheses using abductive reasoning about genes encoding ‘orphan’ enzymes (proteins which do not have a known corresponding gene) in yeast (*S. cerevisiae*). The HyBrow (Hypothesis Browser) system [32,33] was developed to evaluate gene and protein-centric hypotheses about the genetic regulation of galactose metabolism in response to environmental cues using a manually curated knowledge base of literature-extracted facts about the galactose metabolism pathway in yeast coupled with a model for hypotheses and rules. With the exception of Adam the Robot Scientist, these tools, as well as HyQue, are rule-based systems applied to the problems of hypothesis generation, revision and evaluation. In general, rule-based systems [34] consist of a collection of rules in a knowledge base (a ‘rule base’) and an interpreter or inference engine to execute rules triggered by input conditions. Advantages of rule-based systems are that the modular nature of rules facilitates their reuse, new rules can be added to improve the scope and performance of rule bases, and that the ability to trace rule executions makes the reasoning of rule-based systems transparent to users [34]. The approaches to hypothesis formulation and reasoning described above have made significant contributions in terms of methods for formally representing biological hypotheses and scientific data, but the implementation of these representation models is typically system-specific and difficult to apply to new domains and integrate with other tools. Using Semantic Web standards and approaches for data integration to tackle these issues is a promising step forward. HyQue also addresses a need at the core of the biologist’s work [35]: given a hypothesis a biologist already has, our system does the difficult work of retrieving and semi-automatically evaluating what we already know (but may not know to be relevant) in the context of a new biological question.

Computational approaches for gene and protein function prediction comprise a significant area of bioinformatics research that has been extensively reviewed (e.g. [36-38]) and for which benchmarking efforts have been developed [39]. Methods for specifically predicting genes that are involved in aging are fewer in number. Li et al. [40] analyzed known longevity genes in *C. elegans* to learn features for predicting candidate genes using a support vector machine classifier, and achieved a precision of 0.85 and recall of 0.73. Freitas et al. [41] used a supervised learning approach to classify DNA repair genes as aging-related or not, and achieved a maximum AUC of 0.83 for the prediction task on a set of ~140 human genes. Data used as input to the classifiers in [40] and [41] included protein-protein interaction (PPI) network properties, Gene Ontology (GO) annotations and gene expression data. Wan and Freitas [42] followed up with an approach using a Bayesian network classifier trained on GO annotation data to predict *C. elegans* genes with a pro- or anti-longevity effect that achieves an accuracy of 0.68. Most recently, a related method [43] was proposed for improving feature selection methods for aging gene prediction models. The models resulting from such classification approaches must be interpreted to infer their biological significance, and can thus be considered as a hypothesis generation aids. In constrast, HyQue is a tool for automatically gathering evidence and using it to quantify support for a given hypothesis using an evaluation model that is directly interpretable.

In this work, we demonstrate the use of HyQue as a scalable, semantic approach to discover new candidate aging-related genes. In addition to identifying aging gene candidates, HyQue correctly identifies known aging-related genes and provides a quantitative measure of the evidence supporting its evaluations. This work is innovative in several respects: we have developed a novel hypothesis evaluation system that takes advantage of the powerful query and data integration capabilities offered by Semantic Web standards and technologies, and applied our system to a unique and high-impact area of translational bioinformatics focused on the biology of aging across model organisms and humans. In so doing, we have also developed a knowledge base of aging-related biological data and ontologies that is publicly available (at http://hyque.semanticscience.org) for extension and re-use.

## Methods

### HyQue system overview and architecture

**Figure 1:**
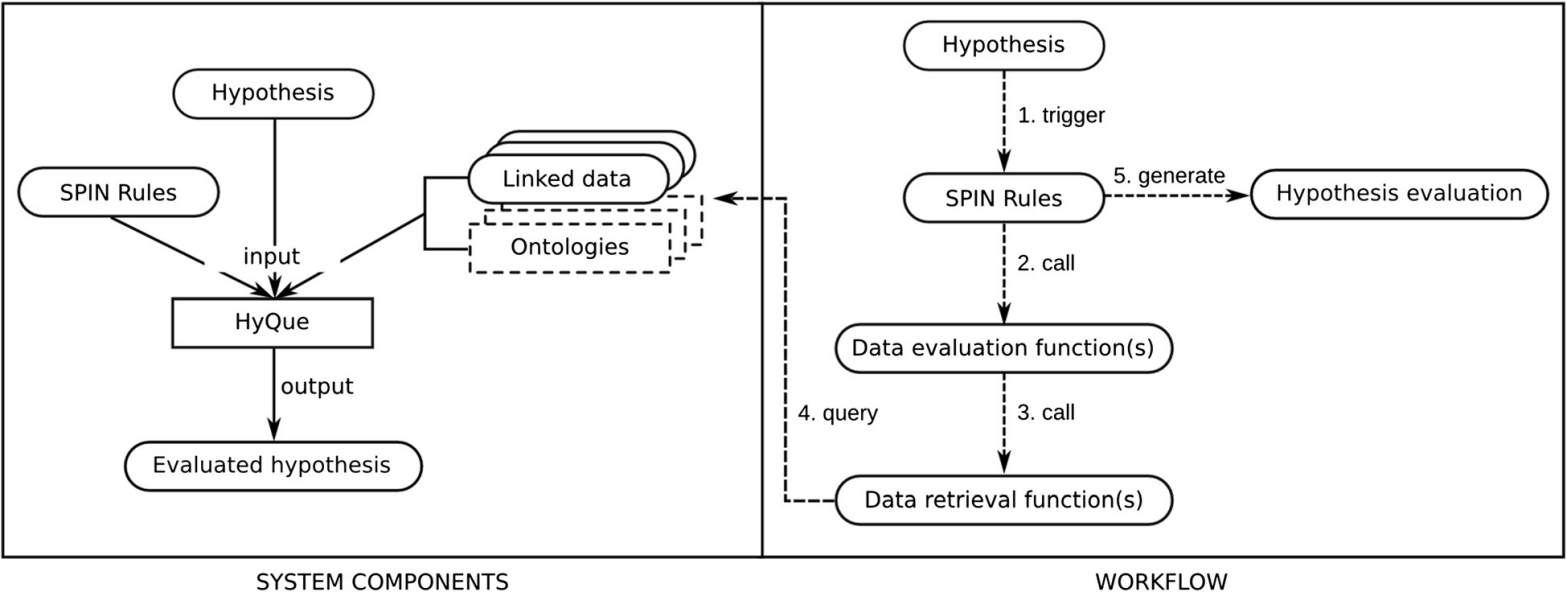
**HyQue system architecture.** HyQue takes as input a hypothesis and a set of SPIN rules, which is executed over a knowledge base, composed of data and ontologies. The hypothesis triggers SPIN rules that retrieve and evaluate relevant data. HyQue produces as output an evaluation including the overall hypothesis score and the individual data contribution scores, as well as links to the SPIN rules used.

HyQue [25] is a rule-based system that retrieves and evaluates evidence relevant to a hypothesis. HyQue rules are specified using SPIN [44], which is a rule model and notation based on SPARQL – the W3C query language for RDF linked data. In the following sections, we describe the HyQue Ontology for hypotheses, events, and hypothesis evaluations, design patterns for rules, data retrieval and data evaluation functions, and explain how HyQue uses these functions to calculate aging-specific event and hypothesis scores. Figure 1 provides an overview of the HyQue system. HyQue takes as input a hypothesis specified in RDF, and a set of domain specific SPIN rules. It executes the SPIN rules to retrieve facts from a knowledge base of relevant RDF data and OWL ontologies, and evaluates the evidence obtained to calculate a score based on support (or lack thereof) that a hypothesis has based on retrieved facts. HyQue generates an RDF output that includes the evaluation, the rules used, and the individual data contribution scores.

### HyQue ontology for hypotheses, events, and evaluations

HyQue uses the HyQue Hypothesis Ontology (HO) [45] to describe a hypothesis and its evaluation. The HO provides a machine understandable vocabulary for describing hypotheses, the output produced by HyQue and the relationships that can hold between them. Specifically, a *hypothesis* is described as a set of one or more *events* that are related to each other by one or more *propositions.* An event is a process that involves one or more participants (e.g. agents, targets), while a proposition relates two or more events through logical operators AND, OR, and XOR. The operators control how the overall hypothesis score is calculated (described below). A *hypothesis evaluation* includes a score for the hypothesis and each of the components of the hypothesis (propositions, events) and their provenance. We use the ovopub [46] for describing the provenance of the evaluation (including links to the input hypothesis and rules used for the evaluation) and linking this provenance to the evaluation itself. An ovopub is a data model for describing an assertion composed of one or more RDF statements along with the metadata of the assertion and the ovopub (including type, creator, and creation time). In this way, it is possible to trace each HyQue evaluation to how and when it was created.

### Design patterns for HyQue functions and rules

HyQue uses two kinds of rules to evaluate a hypothesis – domain specific rules that are triggered by the type(s) of the events described in the hypothesis (e.g. ‘gene induction’, ‘aging’) and system rules which are triggered by the creation of output by the domain specific rules in combination with the operators that relate events in the hypothesis to calculate an overall hypothesis score. HyQue domain specific rules consist of data evaluation functions and data retrieval functions. A data retrieval function executes a SPARQL query over a specified linked data source to obtain statements about an entity of interest (that is specified in the hypothesis). A data evaluation function evaluates the result of a data retrieval function in the context of the biological domain associated with the hypothesized event. Specifically, data evaluation functions call one or more data retrieval functions, assess the retrieved data, and then return a Boolean or numeric value to quantify the assessment. Event scores are calculated by aggregating the output of individual data evaluation functions into a single evaluation score. In this way, HyQue can incorporate contradictory facts into its scoring scheme – if HyQue retrieves data that refutes a hypothesis, this will be taken into account alongside any supporting data, and the aggregated score from the data evaluation functions will be lower than if the refuting fact was not retrieved by the system. Data evaluation and event scoring functions are combined in a rule associated with specific event type(s).

HyQue system rules automatically generate proposition and overall hypothesis evaluation scores from individual event scores generated by the domain rules. These scores are calculated in a bottom-up procedure, in which first event scores are calculated, followed by the proposition scores, and finally the overall hypothesis score. For a proposition that specifies events related by the AND operator, HyQue calculates the proposition score by taking the mean of the individual event scores. For a proposition that specifies events related by an OR operator, HyQue takes the maximum event score as the proposition score. For a proposition that describes a single event (with the XOR operator) the event score is assigned as the proposition score. This procedure is iteratively repeated to calculate the overall hypothesis score. As each score is calculated HyQue generates statements linking the score to the function(s) used to calculate the score, thereby ensuring that provenance of each part of HyQue’s evaluation process is recorded in the evaluation itself.

### Integrating experimental data and annotations about aging in *C. elegans*

HyQue evaluates the role of C*. elegans* genes in aging using a variety of data sources including existing curated databases and raw data, terminologies and ontologies. In the following sections, we describe how we prepared each of the seven data sources for use in HyQue, and related data analysis and transformation processes used. All data sets created in this work are freely available at http://hyque.semanticscience.org.

### Linking aging data on the Semantic Web

**Figure 2:**
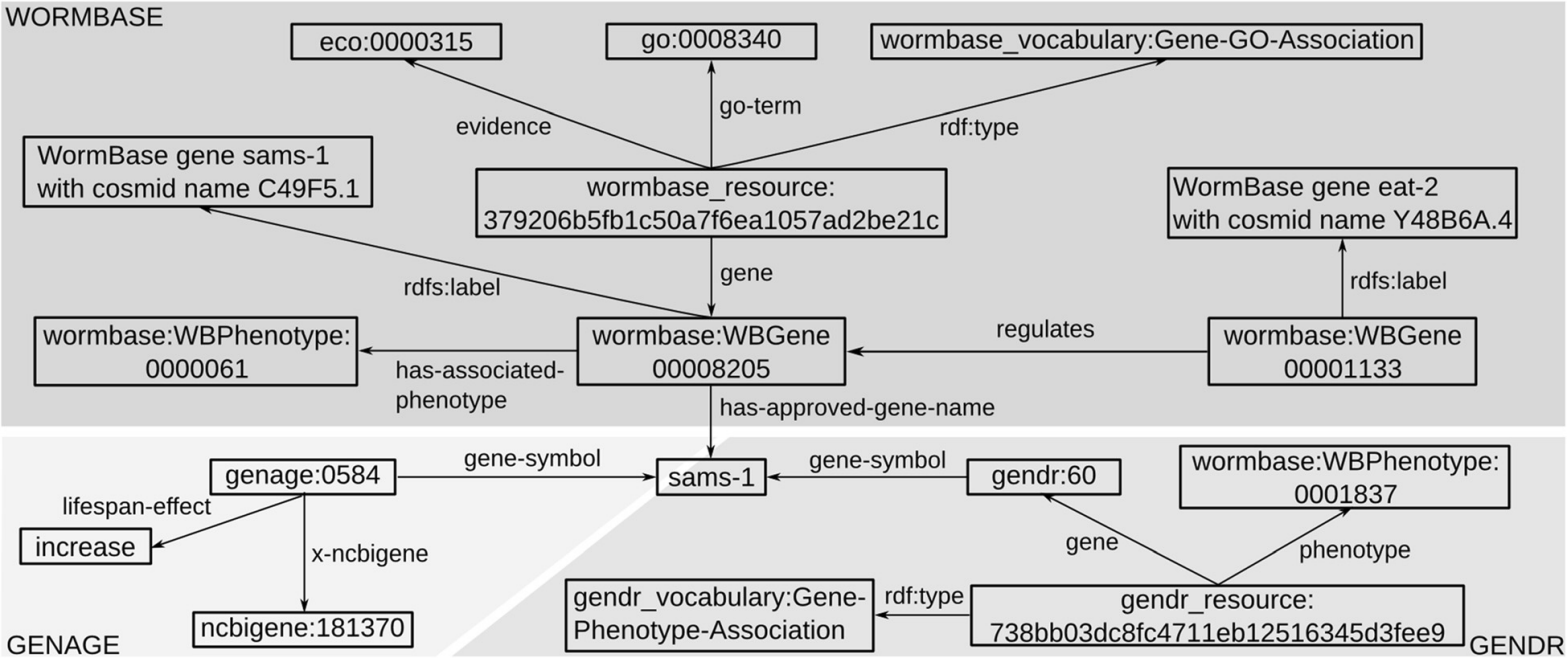
**Statements about the sams-1 gene in Bio2RDF versions of WormBase, GenAge and GenDR, showing how the datasets are connected to each other as well as existing Bio2RDF resources, such as the Gene Ontology (GO).** WormBase includes phenotypes, GO associations, and regulatory relationships for *C. elegans* genes. GenAge includes lifespan effects and cross references to NCBI Gene, as well as other databases. GenDR includes DR-induced gene-phenotype associations. Predicate namespaces correspond to the dataset the predicate occurs in e.g. all WormBase dataset predicates are in the namespace ‘http://bio2rdf.org/wormbase_vocabulary’.

A number of databases dedicated to cataloguing genes that regulate the biological processes of aging have recently been developed, including the GenAge and GenDR databases developed by the Human Ageing Genomic Resources (HAGR) group [47] and the human-curated WormBase database [48]. GenAge describes genes that are known to affect longevity and aging [47], while GenDR describes genes that confer lifespan extension under dietary restriction or whose expression is found to be significantly different under dietary restriction across multiple studies [13]. WormBase annotates *C. elegans* genes with genetic and protein sequence data, known phenotypes and their roles in biological pathways, including those specific to aging processes, as well as links to the literature. As of release WS235, WormBase maintains records for 48,231 genes including protein-coding genes, genes that encode a variety of RNA transcripts (including tRNA, rRNA, sRNA, siRNA etc.) and pseudogenes that do not encode a currently known functional transcript. While the function of pseudogenes has not yet been determined, they may well come to have a known function as a result of new experimental data and are thus valid subjects for evaluation by HyQue. It is not possible to automatically query across these independently maintained resources to collect all the data they contain about a given gene. To enable integration of these databases as evidence sources for HyQue, we generated linked data [49] versions of each. We use the Resource Description Framework (RDF) [50] and Bio2RDF best practices [20] to facilitate dataset interoperability and querying. At the core of the Bio2RDF approach is the use of Uniform Resource Identifiers (URIs) for consistently naming entities and the relationships that hold between them. Using the Bio2RDF approach for data integration ensures that an entity is automatically assigned the same identifier in every dataset that contains statements about it, such that queries across multiple datasets using the same identifier (query federation) will retrieve all statements about a given entity. For example, in the NCBI Gene Bio2RDF dataset, the identifier for the gene sams-1 is http://bio2rdf.org/ncbigene:181370. This same identifier is used in the GenDR Bio2RDF dataset to assert a cross-reference relationship to the GenDR gene identifier for sams-1. In this way, one can query for statements about the sams-1 gene in the NCBI Gene Bio2RDF dataset and the GenDR Bio2RDF GenDR dataset using a single identifier, thereby integrating these datasets. Figure 2 shows partial records in the Bio2RDF versions of WormBase, GenAge and GenDR for the gene sams-1.

### Linked open data relevant to the biology of aging

Relevant sources of data currently available in Bio2RDF (Release 2) include release 9.0 of the iRefIndex database of experimentally determined protein-protein interactions (PPIs) [51], and the Gene Ontology Annotations (GOA) database of protein function, process and cellular location annotations [52] processed in 2012. As described below, these datasets are used in concert with the linked data versions of GenAge, GenDR and WormBase by HyQue to retrieve data about PPIs and functional annotations for *C. elegans* genes. HyQue uses OpenLifeData SPARQL endpoints to access these Bio2RDF datasets. OpenLifeData is a project to provide user interfaces and application programming interfaces to Linked Open Data in the life sciences domain. OpenLifeData enriches Bio2RDF and other RDF data projects to OWL expressivity, implements rich HTTP content-negotiation, and utilizes query-rewriting to resolve OpenLifeData IRIs and SPARQL queries against SPARQL endpoints. It has recently been used to automatically expose SADI semantic web services in a data driven manner to facilitate discovery and reuse of Linked Data [53].

### Gene expression data and analysis

Next-generation sequencing technologies (NGS) measure system-wide gene expression changes under varying experimental conditions. We searched the NCBI’s Gene Expression Omnibus (GEO) database and the literature for gene expression datasets from experiments that targeted biochemical pathways in *C. elegans* and resulted in an extension of lifespan. We identified 20 datasets where different treatments induce *C. elegans* to live longer, and selected for further analysis those datasets obtained with Illumina NGS technology, discarding datasets that did not include base quality scores. Through this process, we identified two relevant RNA-seq datasets – GEO:GSE39574 and GEO:GSE36041. The GSE39574 dataset quantifies changes in gene expression when the transcription factor unc-62 (known to regulate lifespan and aging [54]) is knocked down. The GSE36041 dataset contains the expression profiles of three *C. elegans* models with impaired IGF-1 signaling (the IGF-1 signaling pathway is a well-characterized regulator of longevity [55]). From the raw data in the GSE39574 and GSE36041 GEO records, we first filtered the reads by quality using FastQC [56] and Trimmomatic [57], and then used TopHat and Cufflinks to maps reads to the *C. elegans* genome and find the differentially expressed genes as described in [58].

**Figure 3:**
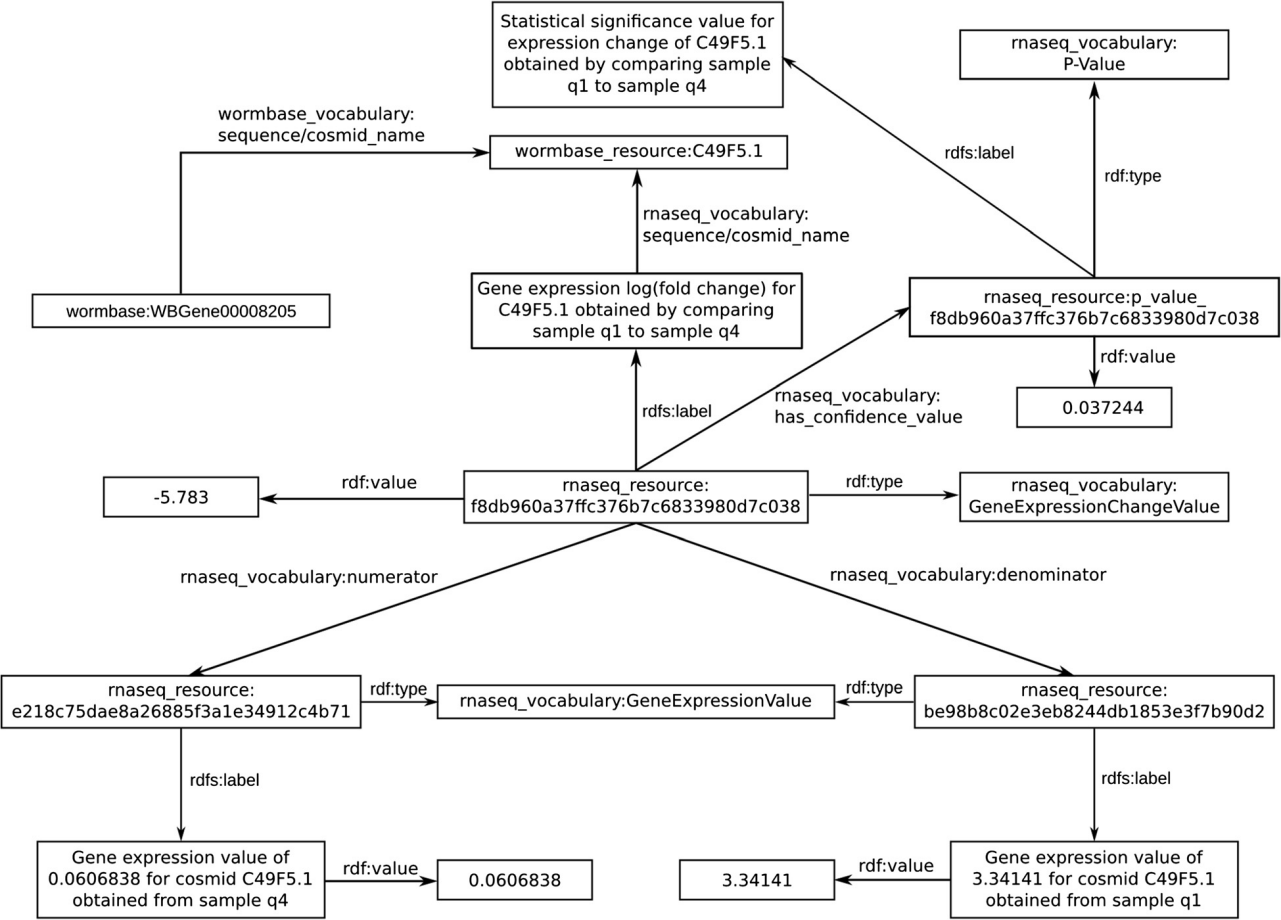
**Example linked data record for a gene expression change value from the GEO:GSE36041 dataset, showing the data the expression change value was derived from, how it is linked to a corresponding p-value, and the WormBase cosmid and gene it was measured for.**

To integrate our genomic data analysis results with the Bio2RDF linked data resources described above, we developed a data model using Bio2RDF best practices to represent the RNA-Seq data analysis results as linked data such that each data item has a unique identifier and links to the values it was derived from and the overall experimental conditions that produced it. Specifically, our model describes experiments and experimental conditions, samples, and the resulting gene expression and gene expression fold change values across samples, as well as the relations that hold between them. It re-uses WormBase identifiers for genes, and associates each gene expression fold change value with its corresponding statistical confidence value (p-value) as well as the gene expression values it is derived from. An example linked data record for a gene expression change value from GEO:GSE36041 is shown in Figure 3.

### Quantifying Gene Ontology annotation co-occurrence

Co-occurrence frequencies of Gene Ontology (GO) terms used for gene and gene product annotations have been analyzed to discover and confirm associations between biological functions and processes [59,60]. In the context of evaluating the role of a given gene in aging, the co-occurrence of its GO annotations with terms related to aging is thus of interest. To measure these co-occurrences, we programmatically calculated the co-occurrence frequency (the total number of times a set of GO terms are used together as annotations of a single entity) of each pair of GO terms in the UniProt GOA database and generated linked data describing these frequencies.

### Tailoring HyQue to the aging domain

**Figure 4:**
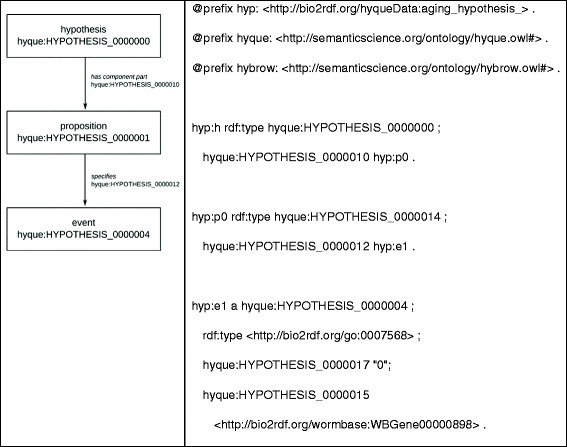
**RDF of HyQue hypothesis that daf-2 is the agent in an aging event.**

To model the hypothesis that a *C. elegans* gene is involved in aging using HO, we used the Gene Ontology term ‘aging’ (GO:0007568) as the event type of interest, where an aging event has as agent a given gene specified with its WormBase Bio2RDF identifier (e.g. ‘daf-2’ is identified as ‘http://bio2rdf.org/wormbase:WBGene00000898’) Figure 4.

We developed domain specific data retrieval and evaluation functions (triggered by an event of type ‘aging’) for investigating the role of *C. elegans* genes in aging. We created 9 domain-specific data evaluation functions (DEFs) for HyQue, each dedicated to assessing different a type of evidence for its contribution to a gene’s involvement in aging, and each evaluating data returned by one or more data retrieval functions (DRFs). The data evaluation functions answer the following questions for a given gene:

1. Does the gene have a human-curated aging- or longevity-associated annotation?
2. Is the gene significantly differentially expressed (under- or over-expressed) when genes that regulate known aging-related pathways are manipulated?
3. Is the gene or a mammalian homolog significantly differentially expressed under dietary restriction across multiple studies?
4. Is the gene’s effect on life-span extension under dietary restriction altered when its expression is manipulated?
5. Does the gene (or its knockdown) have the extended or shortened lifespan phenotype in WormBase?
6. Does the gene have aging-related functional annotations, where the annotation is derived from experimental evidence?
7. Does the gene encode a protein that interacts with other proteins with aging-related functional annotations?
8. Does the gene interact with other genes that extend or shorten lifespan?
9. Does the gene have functional annotations that co-occur with aging-related functional annotations?

The output of each evaluation function call is used to calculate a quantitative score for each event, which are used to generate overall hypothesis scores (see below). We will now describe two data evaluation functions, DEF1 and DEF7, and the DRFs that they call, as well as example results.

**Figure 5:**
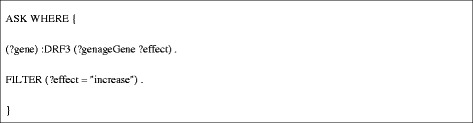
**SPARQL query used for DEF1.**

**DEF1** assesses whether a *C. elegans* gene has been annotated with a role in aging or longevity by calling **DRF3** to query the Bio2RDF GenAge dataset for any human curated annotations on life span. It processes the retrieved data, and returns TRUE if the life span effect retrieved for a gene is “increase”, and FALSE otherwise (Figure 5). DEF1 returns TRUE for the sams-1 gene, which is converted to an evaluation score of 1.

**Figure 6:**
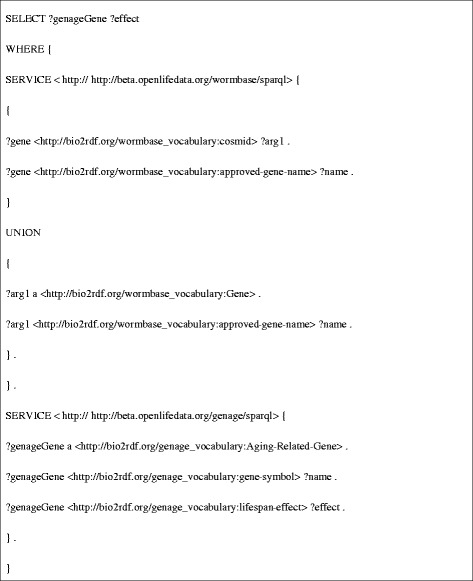
**SPARQL query used for DRF3.**

**DRF3** retrieves a gene’s approved gene name from the Bio2RDF WormBase dataset, and uses this name to query the Bio2RDF GenAge dataset for its effect on lifespan (Figure 6). DRF3 results for the gene sams-1 are shown in Table 1.

**Table 1:** **Results of DRF3 to retrieve a sams-1 lifespan effect from GenAge**

**GenAge gene identifier**	**Lifespan effect**
genage:0584	“increase”

**Figure 7:**
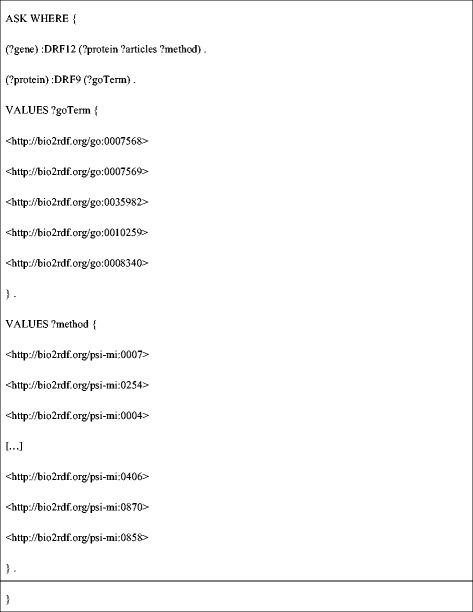
**SPARQL query for DEF7.**

**DEF7** evaluates whether the gene product is associated with any other aging-linked proteins by first calling **DRF12** to retrieve PPIs involving protein products of the gene of interest, and then calling **DRF9** to retrieve GO process annotations for the interacting proteins. It processes the retrieved data and returns TRUE if the interacting protein’s GO annotation is related to aging processes (specifically: ‘aging’ – GO:0007568; ‘cell aging’ – GO:0007569; ‘age-dependent behavioral decline’ – GO:0035982; ‘multicellular organismal aging’ – GO:0010259; ‘determination of adult lifespan’ – GO:0008340) and if the experimental method associated with the PPI is one of a set of high-confidence detection methods (only a subset are shown below), and FALSE otherwise (Figure 7). For the sams-1 gene, DEF7 returns FALSE, which is converted to an evaluation score of 0. The complete list as well as a description of each of the PPI detection methods used as VALUE filters in DEF7 is provided in Additional file 1: Table S9.

**Figure 8:**
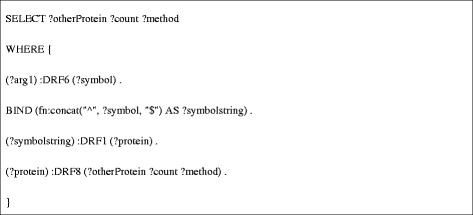
**SPARQL query used for DRF12.**

**Figure 9:**
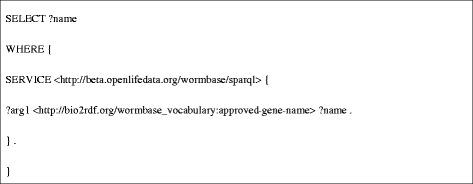
**SPARQL query used for DRF6.**

**Figure 10:**
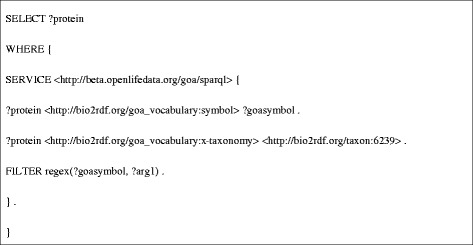
**SPARQL query used for DRF1.**

**Figure 11:**
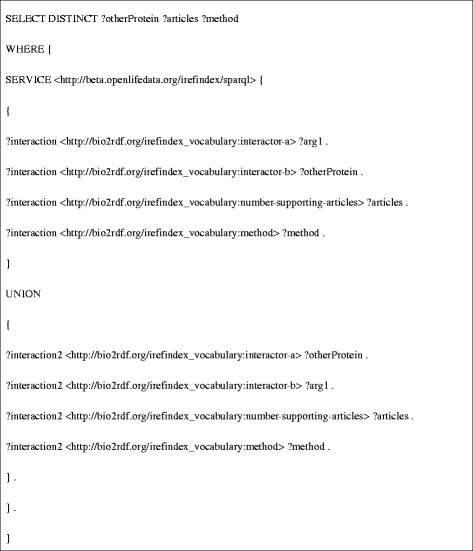
**SPARQL query used for DRF8.cph.**

**DRF12** (Figure 8) requires the coordination of several Bio2RDF data sets, and thus is composed of calls to three other data retrieval functions. Specifically, because iRefIndex uses UniProt identifiers to describe protein-protein interactions, this data retrieval function must retrieve the UniProt identifier for the protein products of a given *C. elegans* gene (specified using its WormBase identifier). To do this it first retrieves the gene name (symbol) for a given WormBase gene identifier by calling **DRF6** (Figure 9). Using the gene symbol, **DRF1** then queries the Bio2RDF GOA dataset for the corresponding UniProt protein identifiers associated with the gene (Figure 10). The resulting UniProt identifiers are used by **DRF8** to query iRefindex for interacting proteins, the experimental method used to detect the interaction and the number of articles reporting the interaction (Figure 11). The results of this query sequence for sams-1 are shown in Table 2.

**Table 2:** **Results of DRF12 to retrieve sams-1 interacting proteins from iRefIndex**

**Protein identifier**	**Number of supporting articles**	**Experimental method identifier**
uniprot:O17680	1	psi-mi:0397
uniprot:O17680	1	psi-mi:0398
uniprot:P48181	1	psi-mi:0676
uniprot:P48181	1	psi-mi:0109
uniprot:P50305	1	psi-mi:0397
uniprot:P50305	1	psi-mi:0398
uniprot:P50306	1	psi-mi:0397
uniprot:P50306	1	psi-mi:0398
uniprot:Q27522	1	psi-mi:0397
uniprot:Q27522	1	psi-mi:0398

In total, HyQue uses 14 data retrieval functions over 7 data sources to collect the evidence used to assess the involvement of a gene in aging. Each function is gene-centric in that it queries a data source to retrieve annotations associated with the *C. elegans* gene specified in the hypothesis. The 14 data retrieval functions (with a brief description) are provided in Additional file 1: Table S10. The complete SPIN RDF representation of all functions and rules is available at the HyQue SPIN Rule GitHub repository [61].

### Evaluating *C. elegans* genes for their role in aging processes

Using the nine data evaluation functions described above, we developed an aging-specific rule triggered when a hypothesis describing an event of the type ‘aging’ (GO:0007568) is input into HyQue. This aging-specific rule calls each of the data evaluation functions described in the previous section, and calculates an overall score for the hypothesis that a given gene is involved in aging. HyQue calculates event scores by computing the sum of the outputs of each of the nine data evaluation functions and dividing this value by the maximum possible score (in this case, 9). For example, a gene that received a score of 1 for 6 of the 9 data evaluation functions would have a normalized score of 6/9 or 0.67, while a gene that satisfied only 3/9 data evaluation functions would have a normalized score of 0.33. This resulting normalized event score is processed by HyQue system rules to automatically generate proposition and overall hypothesis scores, using the logical operators specified for propositions as described above.

We executed the aging rule and functions over each of the 48,231 genes identified in WormBase using a Java implementation of HyQue that uses Jena 2.6.11 and the SPIN API 1.2.1. Using a machine with an Intel i7 quad core processor and 4GB of RAM, processing all 48,231 genes required approximately 48 hours of computing time.

## Results and discussion

### WormBase, GenAge, and GenDR Bio2RDF datasets

The WormBase Bio2RDF dataset (built from Release WS235) contains 20,016,596 statements about 33 types of entities, with 41 relations between those types [62]. In addition to its own native identifiers, the WormBase dataset uses Gene Ontology (GO) for process/function annotations and PubMed identifiers for publications. It also uses the Evidence Codes Ontology (ECO) to specify the type of evidence that is the source of *C. elegans* gene-GO associations. The GenAge Bio2RDF dataset contains 63,474 statements about 16 types, with 42 relations [63]. The GenAge dataset uses NCBI Gene, Ensembl, UniProt, NCBI Taxonomy and PubMed identifiers for genes, proteins, species and publications, respectively. The GenDR Bio2RDF dataset contains 11,081 statements about 15 types, with 34 relations [64]. The GenDR dataset uses NCBI Gene, WormBase, NCBI Taxonomy, and PubMed identifiers for genes, phenotypes, species, and publications, respectively.

### High scoring genes regulate aging in *C. elegans*

Of the 48,231 *C. elegans* genes evaluated by HyQue for their role in aging, the sams-1 gene received the highest score of 0.89 and 7 genes – cco-1, drr-1, jnk-1, pha-4, sgk-1, sir-2.1 and unc-62 – received a score of 0.78. Table 3 lists the genes with their WormBase identifier and gene symbol, as well as the HyQue data evaluation functions that contributed to their high evaluation scores (where the function identifier corresponds to those in the list above). All of these genes have been reported in the literature to regulate longevity (see references in PMID column of Table 3).

**Table 3:** **8** ***C. elegans*** **genes that received the highest HyQue evaluations for their role in aging, the PubMed identifiers of papers describing their roles in regulating longevity, and the data evaluation functions that contributed to their scores**

**WormBase identifier**	**Symbol**	**Score**	**PMID**	**Satisfied data evaluation function**								
				**1**	**2**	**3**	**4**	**5**	**6**	**7**	**8**	**9**
WBGene00008205	sams-1	0.89	16103914	✓	✓	✓	✓	✓	✓		✓	✓
WBGene00000371	cco-1	0.78	21215371	✓	✓			✓	✓	✓	✓	✓
WBGene00009741	drr-1	0.78	16103914	✓	✓		✓	✓	✓		✓	✓
WBGene00002178	jnk-1	0.78	15767565	✓	✓			✓	✓	✓	✓	✓
WBGene00004013	pha-4	0.78	19239417		✓		✓	✓	✓	✓	✓	✓
WBGene00004789	sgk-1	0.78	15068796	✓	✓			✓	✓	✓	✓	✓
WBGene00004800	sir-2.1	0.78	21938067	✓			✓	✓	✓	✓	✓	✓
WBGene00006796	unc-62	0.78	17411345	✓	✓			✓	✓	✓	✓	✓

**Figure 12:**
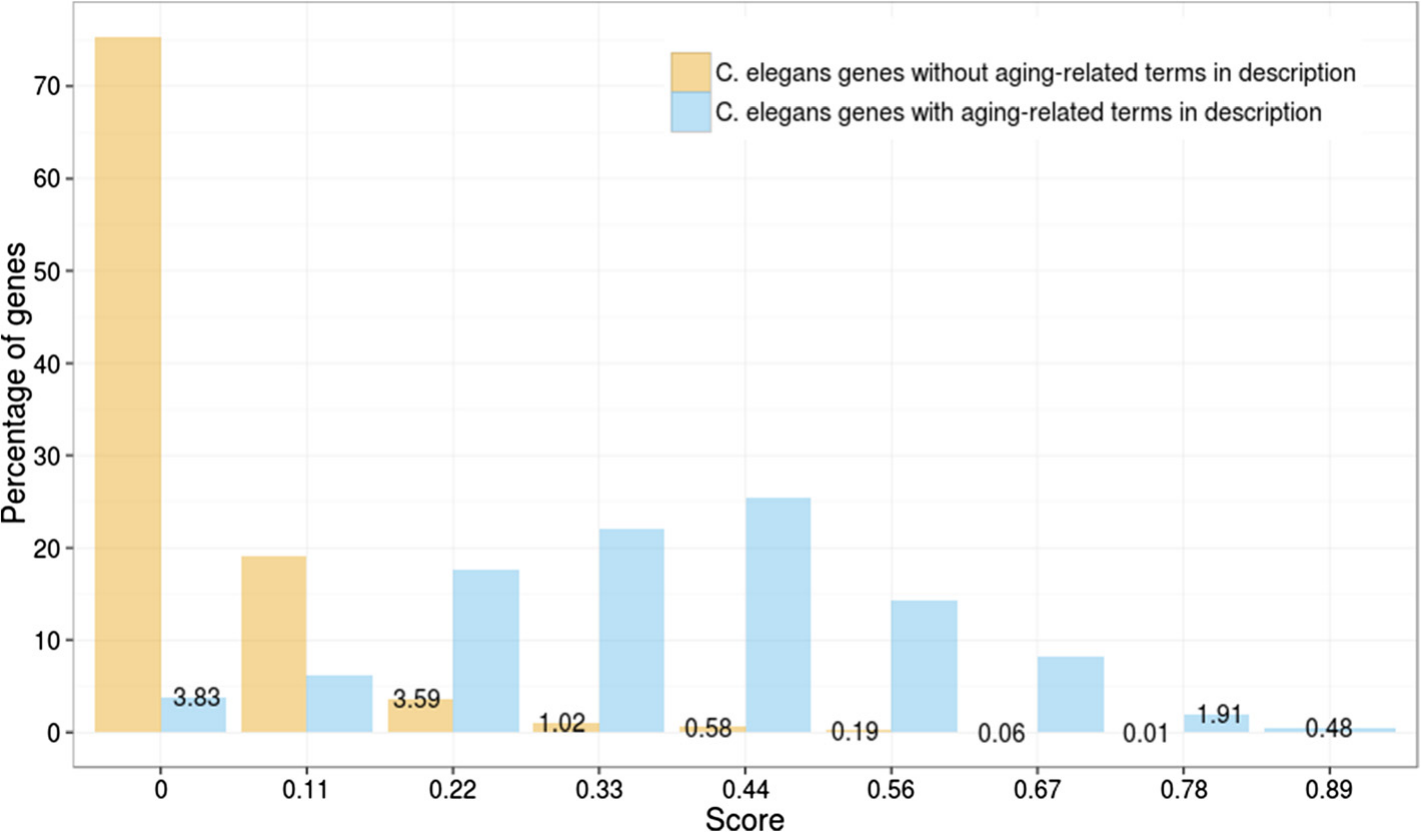
**The HyQue score distribution of** ***C. elegans*** **genes without aging-related terms in their WormBase description is significantly different from that of the scores of 209 genes with aging-related terms in their WormBase descriptions (Kolmogorov-Smirnov test p<2.2x10** ^**−16**^ **).**

We also compared HyQue’s evaluations of genes that would be expected to receive a high score to its evaluation of all other genes, based on a naïve analysis of the gene descriptions in WormBase. Specifically, we queried the Bio2RDF Wormbase dataset for genes that have at least one of the following terms in their WormBase description: “aging”, “lifespan”, “life span” and “longevity”, which returned a set of 209 genes. The distribution of HyQue scores for this set of genes is significantly different from the distribution of HyQue scores for all other *C. elegans* genes (Kolmogorov-Smirnov test p < 2.2x10^−16^; Figure 12). The score distribution of *C. elegans* genes without aging-related terms in their description is heavily left skewed, with 0 being the most frequently assigned score. In contrast, of the 209 genes with aging-related terms in their description, the most frequently assigned score is 0.44 and >50% are assigned that score or higher (comparatively, < 1% of all other genes have a score of 0.44 or higher).

### HyQue identifies candidate aging-related genes in *C. elegans*

The analysis of HyQue scores for genes with aging-related terms in their descriptions provided evidence that HyQue can discern aging-related genes from non-aging-related genes. Thus, we analyzed HyQue evaluations to find the highest scoring genes that do not have an existing aging- or longevity-associated annotation in WormBase or GenAge (i.e. those genes that do not have a scoring contribution from DEF1 or DEF5), indicating that their involvement in aging is not well characterized. There are 31 such genes, all given a score of 0.44 by HyQue. Table 4 lists these genes with the data evaluation functions contributing to their overall score. A closer examination of these 31 genes revealed that there are 7 ‘true positive’ cases (marked with a * in Table 4). Specifically, these 7 genes have WormBase human-readable descriptions that directly implicate them as aging/longevity associated genes and they are annotated with the WormBase ‘life span variant’ phenotype. daf-21 (WBGene00000915) encodes an hsp90-family molecular chaperone known to regulate dauer formation [65], and its RNAi-induced under-expression reduces age-1 modulated lifespan [66]. fkb-3 (WBGene00001428) encodes a peptidylprolyl cis/trans isomerase and its expression is positively regulated by the daf-2 pathway and daf-16 FOXO transcription factor activity [67]. gcy-18 (WBGene00001543) encodes a guanyl cyclase crucial for wild-type thermotaxis [68]. gcy-18 expression is induced in daf-2/daf-16 double mutants, and its knockout by RNAi extends lifespan [69]. hsp-1 (WBGene00002005) encodes heat shock protein hsp70A and its RNAi reduces lifespan in an age-1 mutant [65]. hsp-12.6 (WBGene00002013) is a stress response gene downstream of daf-16. hsp-12.6 expression is increased in daf-2 mutants [11,69] and its silencing by RNAi reduces lifespan by approximately 25% [9]. ikb-1 (WBGene00002069) deletion mutants also have a shortened lifespan [70] and ikb-1 function may be related to DNA damage response [71]. pes-2.1 (WBGene00003977) expression is down-regulated in daf-2 loss-of-function mutants, and RNAi targeting of pes-2.1 increases *C. elegans* lifespan [69]. Three other candidate genes – ges-1 (WBGene00001578), mtl-1 (WBGene00003473) and sod-5 (WBGene00007036) – have the ‘life span variant’ phenotype but their roles in aging/longevity are not characterized and their descriptions in WormBase do not specify whether they extend or shorten lifespan. For ges-1 and mtl-1, the source data cited in WormBase as evidence for the ‘life span variant’ phenotype is from Murphy et al. [69], a study investigating the effect of genes downstream from the daf-16 transcription factor on lifespan. For ges-1, the data indicates that there is not a significant change in lifespan associated with its RNAi diminished expression. For mtl-1, there is data indicating a change in lifespan when its expression is diminished by RNAi, as well as data indicating no change in lifespan. For neither gene is there conclusive evidence presented showing that a change in its expression affects lifespan. The source data for the sod-5 'life span variant' phenotype is from McElwee et al. [72], a study investigating transcriptional changes in expression of daf-16 downstream targets. McElwee et al. provide experimental data indicating that sod-5 RNAi increases lifespan and frequency of dauer formation in daf-2(e1370) mutants. Based on this, it may be that the WormBase phenotype annotation for sod-5 should be updated to more specifically indicate its lifespan-increasing effects.

**Table 4:** **31 highest scoring** ***C. elegans*** **genes that received HyQue evaluation scores for their role in aging without existing aging-related annotations, and the data evaluation functions that contributed to their scores**

**WormBase identifier**	**Symbol**	**Satisfied data evaluation function**								
		**1**	**2**	**3**	**4**	**5**	**6**	**7**	**8**	**9**
WBGene00000252	bli-2		✓					✓	✓	✓
WBGene00000255	bli-5		✓				✓		✓	✓
WBGene00000262	bra-1		✓					✓	✓	✓
WBGene00000479	cgh-1						✓	✓	✓	✓
WBGene00000915*	daf-21						✓	✓	✓	✓
WBGene00001165	efn-4		✓					✓	✓	✓
WBGene00001428*	fkb-3		✓				✓		✓	✓
WBGene00001543*	gcy-18		✓				✓		✓	✓
WBGene00001578	ges-1		✓				✓		✓	✓
WBGene00001746	gsk-3		✓					✓	✓	✓
WBGene00001824	hbl-1		✓				✓		✓	✓
WBGene00001974	hmg-4		✓					✓	✓	✓
WBGene00001979	hmp-2		✓					✓	✓	✓
WBGene00002005*	hsp-1				✓		✓		✓	✓
WBGene00002013*	hsp-12.6		✓		✓				✓	✓
WBGene00002069*	ikb-1		✓		✓				✓	✓
WBGene00002881	let-756		✓					✓	✓	✓
WBGene00003029	lin-44		✓					✓	✓	✓
WBGene00003058	lov-1		✓					✓	✓	✓
WBGene00003210	mel-28						✓	✓	✓	✓
WBGene00003473	mtl-1		✓				✓		✓	✓
WBGene00003497	mup-4		✓					✓	✓	✓
WBGene00003977*	pes-2.1		✓				✓		✓	✓
WBGene00004392	rnr-2						✓	✓	✓	✓
WBGene00004765	sel-8		✓					✓	✓	✓
WBGene00006789	unc-54		✓					✓	✓	✓
WBGene00007036	sod-5		✓				✓		✓	✓
WBGene00016140	rpb-2						✓	✓	✓	✓
WBGene00017830	rpb-8		✓					✓	✓	✓
WBGene00020100	mks-1		✓				✓		✓	✓
WBGene00021334	vps-4		✓					✓	✓	✓

* = true positive.

### Gene Ontology annotation enrichment in candidate aging-related genes

We used FUNC [73] to analyze the 31 candidate genes for significantly enriched biological function and process annotations from GOA, and found that the enriched terms are consistent with role of these genes in aging^a^. Relevant enriched biological process and molecular function terms are listed in Tables 5 and 6, respectively. Additional file 1: Tables S11 and S12 list all significantly enriched GO biological processes and molecular functions, respectively, and their associated p-values.

**Table 5:** **GO biological process annotations enriched in the set of 31 C. elegans candidate aging-related genes identified by HyQue that are consistent with their potential role in aging**

**Biological process**	**GO identifier**	**P-value**
Determination of adult lifespan	go:0008340	2.7 x 10^-11^
Larval foraging behavior	go:0035177	1.1 x 10^-2^
Multicellular organismal reproductive process	go:0048609	1.3 x 10^-2^
Superoxide metabolic process	go:0006801	1.7 x 10^-2^
Inositol lipid-mediated signaling	go:0048017	1.9 x 10^-2^
Deoxyribonucleoside diphosphate metabolic process	go:0009186	2.5 x 10^-2^
Protein folding	go:0006457	2.6 x 10^-2^
Nematode larval development	go:0002119	4.6 x 10^-3^
Multicellular organismal protein metabolic process	go:0044268	5.7 x 10^-3^
Response to heat	go:0009408	6.7 x 10^-3^

**Table 6:** **GO molecular function annotations enriched in the set of 31 C. elegans candidate aging-related genes identified by HyQue that are consistent with their potential role in aging**

**Molecular function**	**GO identifier**	**P-value**
Structural constituent of collagen and cuticulin-based cuticle	go:0042329	2.9 x 10^-4^
Fibroblast growth factor receptor binding	go:0005104	5.2 x 10^-3^
Superoxide dismutase activity	go:0004784	1.2 x 10^-2^
Growth factor activity	go:0008083	2.1 x 10^-2^
Transcription coactivator activity	go:0003713	3.6 x 10^-2^

### Distribution of HyQue scores and data evaluation function score contributions

The overall HyQue score distribution of all *C. elegans* genes is shown in Table 7. As described above, 8 genes received very high scores, indicating that HyQue retrieved evidence from many sources that they are involved in aging. The majority received a score of 0, indicating that HyQue found no evidence in the data sources it queried that those genes are involved in aging. HyQue captures the individual score contributions of each data evaluation function for each *C. elegans* gene, and using this data we measured the frequencies with which each function was satisfied across all *C. elegans* genes. Table 8 shows the frequency with which each of the 9 data evaluation functions were satisfied across all *C. elegans* genes.

**Table 7:** **HyQue score distribution for 48,231** ***C. elegans*** **genes**

**Score**	**Number of genes**
0.89	1
0.78	7
0.67	46
0.56	122
0.44	333
0.33	537
0.22	1759
0.11	9200
0	36226

**Table 8:** **Frequency with which each data evaluation function was satisfied across all 48,231 C. elegans genes**

**Data evaluation function**	**Satisfied frequency**	**Proportion (Frequency/# of** ***C. elegans*** **genes)**
DEF1	317	6.6x10^-3^
DEF2	6406	1.3x10^-1^
DEF3	1	2.1x10^-5^
DEF4	55	1.1x10^-3^
DEF5	699	1.4x10^-2^
DEF6	876	1.8x10^-2^
DEF7	135	2.8x10^-3^
DEF8	1216	2.5x10^-2^
DEF9	6899	1.4x10^-1^

No *C. elegans* genes achieved the maximum possible normalized HyQue score of 1, because no single gene had all features required to satisfy the 9 data evaluation functions used in its evaluation. More specifically, only one *C. elegans* gene satisfied DEF3, which asked if a given gene or its homolog was significantly differently expressed under dietary restriction across multiple studies, using the Bio2RDF GenDR dataset as a source. GenDR includes the homologs of 99 model organism genes, but only three of these had entries in the GenDR list of DR affected genes from multiple studies, and of those only one was a homolog of a *C. elegans* gene. This dataset will be important for future applications of HyQue, however, as we extend its application to evaluating the role of mammalian genes in aging in a manner similar to the approach described here. Also, if the GenDR database is expanded to include non-mammals, then it may become increasingly relevant for a wider set of aging-related hypotheses.

The most frequently occurring combination of satisfied data evaluation functions (used to generate the overall HyQue evaluation score) for the 31 candidate aging-related genes is DEF2, DEF7, DEF8 and DEF9. Based on the overall frequencies of these functions being satisfied (Table 8) across all *C. elegans* genes, the likelihood of observing this combination by chance for a single gene is just 1.3x10^−6^. Considering that there are 48,231 genes in WormBase, less than one gene in this set would have this combination by chance. This, as well as the distribution of HyQue scores for genes with aging-related terms in their descriptions validates the HyQue approach to assessing biological hypotheses. Genes that have accumulated more biological evidence (as determined by the execution of data retrieval and evaluation functions) are better candidates for satisfying the hypothesis that they are involved in aging and thus receive a higher evaluation score. The set of 209 genes with aging-related terms in their descriptions likely do not comprise *all* genes that have a role in aging, but the occurrence of aging-related terms in these genes’ descriptions implicates them as aging-related genes. HyQue was therefore expected to evaluate them as such and assign them higher evaluation scores than would be expected by chance, as was observed.

The scoring system used by HyQue to evaluate a gene’s role in aging is one of many possible variations, and will improve over time. For example, currently all evidence types are assigned the same weight, and so the presence or absence of any evidence equally affects HyQue’s final evaluation. However, some evidence, such as experimentally measured changes in gene expression, may have more validity in confirming or refuting a hypothesis. Increasing the score contributed by gene expression data so that its value affects a final score more than a less powerful data source, such as a one-step-removed genetic interaction, could reflect this evidence quality. It may also be that different scientists will come to view the same evidence with varying confidence, and HyQue’s evaluation functions can evolve over time to reflect these shifts in perspective. HyQue’s automatically generated provenance of hypothesis evaluations is useful in this context, as it makes it possible to determine exactly how a hypothesis achieved a given score, by following links to evaluation rules and individual data score contributions. Data retrieval and data evaluation are separated to facilitate the re-use of data retrieval functions for different hypothesis types, and also in an attempt to future proof HyQue functions in the event that a data source changes, or a data evaluation criteria changes over time. Maintaining data retrieval and evaluation functions separately means that either can be updated without requiring that the other be changed.

Performance evaluation measures such as veracity [74], recently proposed as an alternative approach to precision and recall for evaluating predictive systems, may also be useful in assessing HyQue’s ability to correctly evaluate hypotheses. Veracity quantifies the performance of systems that predict features such as a chemical’s toxicological activity by considering what proportion of a set of entities that are input into the system should ideally fall into each of the possible predicted categories, and comparing the observed proportions to this ideal. In other words, veracity quantifies the confidence level associated with a given prediction, in that we can have more confidence in predictions that more closely follow the ideal distribution. Using veracity to assess HyQue’s evaluations of *C. elegans* genes for their involvement in aging would require an ideal distribution of scores, which would in part require verification of each gene’s role (or lack thereof) in aging. Such an assessment may be possible in the near future.

### Candidate aging-related genes are good targets for future experiments

We have demonstrated that HyQue is able to correctly identify known aging/longevity-related genes in *C. elegans* by evaluating a variety of evidence types from multiple sources, and can also identify candidate aging and longevity-related genes whose effect on these biological processes are not yet well-characterized. Indeed, the 24 candidate genes (not including the 7 true positives of the 31 candidates) are promising targets for future research to uncover their effects on lifespan. The HyQue data evaluation functions that were not satisfied for each of these genes can be used as a guide for future experimental designs. For example, given that experimental data about the expression of gsk-3 under dietary restriction is not currently available in GenDR, an experiment could be performed to obtain this data. Of the 24 candidate genes lacking direct links to aging/longevity, the majority has known functions related to development, stress response (including protection against environmental stresses such as heat and oxidative damage) and reproductive behavior in *C. elegans*. Human orthologs [75] of several of these genes are also responsible for neurodegenerative disease phenotypes. For example, polymorphisms in a human ortholog of let-756, FGF20, are risk factors for Parkinson’s disease [76,77]. Similarly, a human ortholog of gsk-3, GSK3B, may also modulate risk for Parkinson’s disease [78] and Alzheimer’s disease [79,80]. Mutations in SOD1, a human ortholog of sod-5 that functions to destroy free superoxide radicals in the body and protect against RNA, DNA and protein damage, are associated with amyotrophic lateral schlerosis (ALS, or Lou Gehrig’s disease). All of these human disorders are associated with shortened lifespan [81-83].

### Limitations

The HyQue framework for hypothesis evaluation is subject to some limitations. Because HyQue relies on external data sources to obtain relevant evidence in support of or refuting a given hypothesis, a change in the content of those sources may affect the evaluation that HyQue assigns a hypothesis. Thus, effort is required to keep track of the status of data sources and re-execute HyQue over a given hypothesis if a source is updated. For example, Bio2RDF is a resource that is developed using many external biological data sources which may be updated at a faster rate than the current Bio2RDF release schedule. As new data relevant to a specific domain becomes available, it may also be required to update hypothesis evaluation rules that are used by HyQue. It is possible to automate some of these processes, but they currently require manual maintenance by HyQue users. Domain specific data evaluation functions are the product of a given user’s perspective on the relevant of a given data source to a hypothesis, and these perspectives may differ or even be contradictory among different HyQue users. HyQue hypothesis evaluations are thus subject to the same biases and potential errors that human researchers encounter. The benefit that HyQue offers in such cases, however, is that regardless of the domain specific evaluation rules that HyQue uses, the provenance of each evaluation it produces is unambiguously represented for both humans and machines, allowing the reasoning behind each hypothesis evaluation to be understood.

### Future work

Future work will involve experimental validation of the 24 candidate genes for their role in aging-related biological processes, as well as continued development of the data retrieval and evaluation functions used by HyQue to assess a gene’s role in aging. Specifically, it is possible to expand the taxonomic reach of HyQue by including evidence from additional model organisms as such data becomes available.

## Conclusions

We have described the application of HyQue, a Semantic Web tool for hypothesis evaluation, to the problem of discovering *C. elegans* genes that affect aging and longevity. We show that HyQue gives positive scores to hypotheses involving genes that are known to regulate aging, and also identified 24 potential aging-related genes that are good candidates for experimental study in this context. HyQue realizes the promise of the Semantic Web [84] to bring relevant knowledge automatically to the fingers of biologists studying complex domains, and to reason over this knowledge for assessing biological hypotheses. With current biological evidence, the functions used to evaluate that evidence, and the outcomes of HyQue evaluations all serialized as Linked Data (available for download at http://hyque.semanticscience.org), it is possible to query and reason over these resources to discover how evidence changes over time, and how this affects prevailing biological hypotheses. HyQue data retrieval and evaluation SPIN functions can also be repurposed for new biological domains, and their availability as linked data whose properties can be computationally queried (for example, to discover functions that satisfy a given criteria or retrieve a certain data type) makes them ideal for re-use.

## Endnote

^a^ p-values were calculated after a FUNC refinement step to remove GO terms that were enriched only because their child terms were enriched.

## Additional file

Additional file 1: Table S9. Protein-protein interaction detection methods used by DEF7 to filter results. **Table S10.** Descriptions of the 14 data retrieval functions used by HyQue, grouped by the dataset queried in the function. **Table S11.** All GO biological process annotations enriched in the set of 31 C.elegans candidate aging-related genes identified by HyQue. **Table S12.** GO molecular function annotations enriched in the set of 31 C. elegans candidate aging-related genes identified by HyQue.
