# Supplementary material for: An evidence-based approach to identify aging-related genes in Caenorhabditis elegans
Source: BMC Bioinformatics. 2015 Feb 7;16(1):40. doi: 10.1186/s12859-015-0469-4 (PMC4339751; doi:10.1186/s12859-015-0469-4)
Supplement: Additional file 1: Table S9. — Protein-protein interaction detection methods used by DEF7 to filter results. Table S10. Descriptions of the 14 data retrieval functions used by HyQue, grouped by the dataset queried in the function. Table S11. All GO biological process annotations enriched in the set of 31 C.elegans candidate aging-related genes identified by HyQue. Table S12. GO molecular function annotations enriched in the set of 31 C. elegans candidate aging-related genes identified by HyQue. [file 12859_2015_469_MOESM1_ESM.doc]

# Additional file

Table S1 Protein-protein interaction detection methods used by DEF7 to filter results

| **Method identifier** | **Name** |
| --- | --- |
| psi-mi:0004 | affinity chromatography technology |
| psi-mi:0006 | anti bait coimmunoprecipitation |
| psi-mi:0007 | anti tag coimmunoprecipitation |
| psi-mi:0012 | bioluminescence resonance energy transfer |
| psi-mi:0019 | coimmunoprecipitation |
| psi-mi:0020 | transmission electron microscopy |
| psi-mi:0040 | electron microscopy |
| psi-mi:0055 | fluorescent resonance energy transfer |
| psi-mi:0067 | light scattering |
| psi-mi:0069 | mass spectrometry studies of complexes |
| psi-mi:0077 | nuclear magnetic resonance |
| psi-mi:0096 | pull down affinity chromatography |
| psi-mi:0107 | surface plasmon resonance |
| psi-mi:0109 | tap tag coimmunoprecipitation |
| psi-mi:0114 | X-ray crystallography |
| psi-mi:0254 | genetic interference |
| psi-mi:0364 | inferred by curator |
| psi-mi:0405 | competition binding |
| psi-mi:0406 | deacetylase assay |
| psi-mi:0410 | electron tomography |
| psi-mi:0411 | enzyme linked immunosorbent assay |
| psi-mi:0415 | enzymatic study |
| psi-mi:0417 | Footprinting |
| psi-mi:0423 | in-gel kinase assay |
| psi-mi:0424 | protein kinase assay |
| psi-mi:0434 | phosphatase assay |
| psi-mi:0435 | protease assay |
| psi-mi:0515 | methyltransferase assay |
| psi-mi:0676 | tandem affinity purification |
| psi-mi:0678 | antibody array |
| psi-mi:0728 | gal4 vp16 complementation |
| psi-mi:0809 | bimolecular fluorescence complementation |
| psi-mi:0826 | X-ray scattering |
| psi-mi:0841 | phosphotransfer assay |
| psi-mi:0858 | immunodepleted coimmunoprecipitation |
| psi-mi:0870 | demethylase assay |

Table S2 Descriptions of the 14 data retrieval functions used by HyQue, grouped by the dataset queried in the function.

| **DRF** | **Description** |
| --- | --- |
| DRF1 | Retrieve *C. elegans* gene product UniProt identifier(s) from GOA – The Bio2RDF UniProt GOA dataset maps gene symbols to UniProt protein identifiers. This DRF retrieves UniProt identifiers of the protein products of a C. elegans gene specified by its gene symbol. |
| DRF2 | Retrieve GO term co-occurrence frequencies from GO term co-occurrences in GOA – We calculated the GO term co-occurrence frequencies for each pair of GO terms used in GOA. This DRF retrieves the frequency of co-occurrence of a given pair of GO terms. |
| DRF9 | Retrieve C. elegans gene product GO process annotations from GOA – The Bio2RDF GOA dataset includes GO process annotations for proteins specified with UniProt identifiers. This DRF retrieves GO annotations for the protein products of a C. elegans gene specified by its WormBase identifier where the UniProt identifiers of the protein products of the gene were retrieved by DRF1. |
| DRF4 | Retrieve C. elegans gene fold change and probability value from NGS GEO datasets – Our analysis of NGS GEO datasets GEO36041 and GEO39574 quantified gene fold change (relative to a control) and associated p-values from experimental data. This DRF retrieves gene fold change values and associated p-values for a C. elegans gene specified by its WormBase identifier. |
| DRF3 | Retrieve C. elegans gene identifier and lifespan effect from GenAge – The GenAge Bio2RDF dataset we generated includes the assigned GenAge identifier and known lifespan effect(s), if any, for C. elegans genes. This DRF retrieves the GenAge identifier and lifespan effect of a given C. elegans gene, specified by its WormBase identifier. |
| DRF11 | Retrieve C. elegans gene DR-associated gene expression change values from GenDR – The Bio2RDF GenDR dataset includes experimentally determined gene expression changes associated with dietary restriction (DR). This DRF retrieves gene expression change values under DR for a C. elegans gene specified by its gene symbol. |
| DRF13 | Retrieve C. elegans gene-phenotype associations from GenDR – The Bio2RDF GenDR dataset contains DR-related gene-phenotype associations. This DRF retrieves the DR-related phenotypes for a C. elegans gene specified by its WormBase identifier. |
| DRF5 | Retrieve C. elegans gene GO annotation and evidence type from WormBase – The Bio2RDF WormBase dataset provides GO annotations and source evidence type (using the ECO ontology) for C. elegans genes. This DRF retrieves the GO annotation and associated evidence type for a C. elegans gene specified by its WormBase identifier. |
| DRF6 | Retrieve C. elegans gene symbol from WormBase – The Bio2RDF WormBase dataset provides the official gene symbols for C. elegans genes. This DRF retrieves the gene symbol for a C. elegans gene specified by its WormBase identifier. |
| DRF7 | Retrieve interacting C. elegans genes with extended or shortened lifespan phenotype from WormBase – The Bio2RDF WormBase dataset includes curated genetic interactions between C. elegans genes. This DRF retrieves any interacting genes that have the WormBase ‘extended lifespan’ or ‘shortened lifespan’ phenotype for a C. elegans gene specified by its WormBase identifier. |
| DRF10 | Retrieve C. elegans gene associated phenotype from WormBase – The Bio2RDF WormBase dataset provides phenotypes associated with C. elegans genes. This DRF retrieves phenotypes associated with a C. elegans gene specified by its WormBase identifier. |
| DRF14 | Retrieve C. elegans gene RNAi-induced phenotypes from WormBase – The Bio2RDF WormBase dataset includes the results of RNAi experiments, including the phenotypes associated with genes whose expression was diminished by RNAi This DRF retrieves the observed phenotypes from RNAi experiments targeting a given C. elegans gene specified by its WormBase identifier. |
| DRF8 | Retrieve gene product interacting proteins and interaction metadata from iRefIndex – The Bio2RDF iRefIndex dataset describes protein-protein interactions (PPIs), associated experimental methods, and number of publications that report a given interaction. This DRF retrieves PPIs involving proteins specified by their UniProt identifier. |
| DRF12 | Retrieve C. elegans gene product interacting proteins from iRefIndex – This DRF retrieves PPIs for the protein products (retrieved by DRF1) of a C. elegans gene (via its symbol as retrieved by DRF6) specified by its WormBase identifier, as well as the experimental method used to detect each PPI and the number of reporting publications (retrieved by DRF8). |

Table S3 All GO biological process annotations enriched in the set of 31 C.elegans candidate aging-related genes identified by HyQue

| **Biological process** | **GO identifier** | **P-value** |
| --- | --- | --- |
| negative regulation of translation | go:0017148 | 0.042 |
| morphogenesis of embryonic epithelium | go:0016331 | 0.034 |
| multi-organism reproductive behavior | go:0044705 | 0.034 |
| engulfment of apoptotic cell | go:0043652 | 0.030 |
| protein folding | go:0006457 | 0.026 |
| mitotic spindle organization | go:0007052 | 0.026 |
| deoxyribonucleoside diphosphate metabolic process | go:0009186 | 0.026 |
| thermosensory behavior | go:0040040 | 0.026 |
| response to cadmium ion | go:0046686 | 0.026 |
| Wnt receptor signaling pathway, regulating spindle positioning | go:0060069 | 0.026 |
| sexual reproduction | go:0019953 | 0.023 |
| cuticle development involved in collagen and cuticulin-based cuticle molting cycle | go:0042338 | 0.024 |
| cytoskeletal anchoring at plasma membrane | go:0007016 | 0.020 |
| regulation of cell adhesion | go:0030155 | 0.020 |
| inositol lipid-mediated signaling | go:0048017 | 0.020 |
| superoxide metabolic process | go:0006801 | 0.017 |
| fibroblast growth factor receptor signaling pathway | go:0008543 | 0.017 |
| tail tip morphogenesis | go:0045138 | 0.016 |
| skeletal muscle myosin thick filament assembly | go:0030241 | 0.014 |
| negative regulation of transforming growth factor beta receptor signaling pathway | go:0030512 | 0.014 |
| regulation of axon extension involved in axon guidance | go:0048841 | 0.014 |
| negative regulation of synapse assembly | go:0051964 | 0.014 |
| gonad development | go:0008406 | 0.014 |
| multicellular organismal reproductive process | go:0048609 | 0.013 |
| negative regulation of cell projection organization | go:0031345 | 0.011 |
| larval foraging behavior | go:0035177 | 0.011 |
| retrograde transport, endosome to Golgi | go:0042147 | 0.011 |
| cell fate specification involved in pattern specification | go:0060573 | 0.011 |
| receptor guanylyl cyclase signaling pathway | go:0007168 | 0.0086 |
| multicellular organismal protein catabolic process | go:0044254 | 0.0086 |
| Hatching | go:0035188 | 0.0073 |
| hermaphrodite genitalia development | go:0040035 | 0.0072 |
| response to heat | go:0009408 | 0.0067 |
| germline cell cycle switching, mitotic to meiotic cell cycle | go:0051729 | 0.0064 |
| multicellular organismal protein metabolic process | go:0044268 | 0.0057 |
| nematode larval development | go:0002119 | 0.0046 |
| regulation of actin cytoskeleton organization by cell-cell adhesion | go:0090138 | 0.0029 |
| apoptotic process | go:0006915 | 0.0019 |
| receptor-mediated endocytosis | go:0006898 | 0.00094 |
| morphogenesis of an epithelium | go:0002009 | 0.00094 |
| cell migration involved in gastrulation | go:0042074 | 0.00093 |
| Locomotion | go:0040011 | 0.00030 |
| ATP catabolic process | go:0006200 | 0.00028 |
| determination of adult lifespan | go:0008340 | 2.2E-11 |

Table S4 GO molecular function annotations enriched in the set of 31 C. elegans candidate aging-related genes identified by HyQue

| **Molecular function** | **GO identifier** | **P-value** |
| --- | --- | --- |
| four-way junction helicase activity | go:0009378 | 0.048 |
| actin filament binding | go:0051015 | 0.041 |
| transcription coactivator activity | go:0003713 | 0.036 |
| ATP binding | go:0005524 | 0.024 |
| ATPase activity | go:0016887 | 0.021 |
| ribonucleoside-diphosphate reductase activity, thioredoxin disulfide as acceptor | go:0004748 | 0.021 |
| growth factor activity | go:0008083 | 0.021 |
| frizzled binding | go:0005109 | 0.015 |
| RNA helicase activity | go:0003724 | 0.013 |
| superoxide dismutase activity | go:0004784 | 0.013 |
| structural constituent of muscle | go:0008307 | 0.013 |
| cadherin binding | go:0045296 | 0.010 |
| unfolded protein binding | go:0051082 | 0.0085 |
| protein kinase binding | go:0019901 | 0.0079 |
| microfilament motor activity | go:0000146 | 0.0052 |
| fibroblast growth factor receptor binding | go:0005104 | 0.0052 |
| protein domain specific binding | go:0019904 | 0.0041 |
| alpha-catenin binding | go:0045294 | 0.0026 |
| structural constituent of collagen and cuticulin-based cuticle | go:0042329 | 0.00029 |
